# Combined effects of neuroticism and negative emotional context on spontaneous EEG dynamics

**DOI:** 10.1093/scan/nsae012

**Published:** 2024-02-09

**Authors:** Michele Deodato, Martin Seeber, Kevin Mammeri, Christoph M Michel, Patrik Vuilleumier

**Affiliations:** Laboratory for Behavioral Neurology and Imaging of Cognition, Department of Fundamental Neurosciences, University Medical School of Geneva, Geneva 1202, Switzerland; Psychology Program, Division of Science, New York University Abu Dhabi, Abu Dhabi, UAE; Functional Brain Mapping Laboratory, Department of Fundamental Neurosciences, Campus Biotech, University of Geneva, Geneva 1201, Switzerland; Laboratory for Behavioral Neurology and Imaging of Cognition, Department of Fundamental Neurosciences, University Medical School of Geneva, Geneva 1202, Switzerland; Swiss Center for Affective Sciences, University of Geneva, Campus Biotech, Geneva 1202, Switzerland; Functional Brain Mapping Laboratory, Department of Fundamental Neurosciences, Campus Biotech, University of Geneva, Geneva 1201, Switzerland; Center for Biomedical Imaging (CIBM), Lausanne and Geneva, Lausanne 1015, Switzerland; Laboratory for Behavioral Neurology and Imaging of Cognition, Department of Fundamental Neurosciences, University Medical School of Geneva, Geneva 1202, Switzerland; Swiss Center for Affective Sciences, University of Geneva, Campus Biotech, Geneva 1202, Switzerland

**Keywords:** neuroticism, EEG microstates, fear, resting state

## Abstract

Neuroticism is a personality trait with great clinical relevance, defined as a tendency to experience negative affect, sustained self-generated negative thoughts and impaired emotion regulation. Here, we investigated spontaneous brain dynamics in the aftermath of negative emotional events and their links with neuroticism in order to shed light on the prolonged activity of large-scale brain networks associated with the control of affect. We recorded electroencephalography (EEG) from 36 participants who were asked to rest after watching neutral or fearful video clips. Four topographic maps (i.e. microstates classes A, B, C and D) explained the majority of the variance in spontaneous EEG. Participants showed greater presence of microstate D and lesser presence of microstate C following exposure to fearful stimuli, pointing to changes in attention- and introspection-related networks previously associated with these microstates. These emotional effects were more pronounced for participants with low neuroticism. Moreover, neuroticism scores were positively correlated with microstate C and negatively correlated with microstate D, regardless of previous emotional stimulation. Our results reveal distinctive effects of emotional context on resting-state EEG, consistent with a prolonged impact of negative affect on the brain, and suggest a possible link with neuroticism.

Highlights

- Exposure to fearful video clips affects subsequent resting-state EEG microstates dynamics.
- Post-emotion rest enhances a topography (class D) previously associated with attention networks, but reduces a topography (class C) associated with default mode and saliency networks.
- Fear-induced effects on these two resting-state classes are further modulated by individual neuroticism scores.
- Temporal parameters of microstates C and D correlate with neuroticism scores regardless of previous emotion events, suggesting a bias favoring introspective functions at the expense of attention processes in neurotics.

## Introduction

Negative emotional events can trigger specific brain responses not only during the events but also have a prolonged impact on neural and behavioral functioning afterward. The nature and significance of these sustained emotional effects remain poorly understood and rarely examined in neuroimaging research (Eryilmaz *et al*., 2011, 2014; Gaviria *et al*., 2021). Moreover, these effects may vary among individuals and reflect differences in emotion regulation or emotion recovery ability (Kuppens *et al*., 2010). In particular, neuroticism is a personality trait characterized as a tendency to experience negative affect and impaired emotional regulation (Suls *et al*., 1998; Lahey, 2009; Kuppens *et al*., 2010) that has been shown to predict a variety of physical and mental illnesses, especially internalizing disorders (e.g. depression, anxiety disorders) (Ormel *et al*., 2004, 2013b; Lahey, 2009). Individuals with high neuroticism use poor coping strategies to deal with psychological distress (e.g. wishful thinking, rumination, avoidance) and experience greater worry in everyday life situations (Suls and Martin, 2005; Servaas *et al*., 2014). Intrusive thoughts and internalizing coping strategies are widely considered a central aspect of the neurotic personality. Specifically, negative self-generated thoughts could represent a major cause for neuroticism (Perkins *et al*., 2015) and the cognitive interference or ‘mental noise’ introduced by these processes could explain a lower cognitive performance of neurotic individuals in attention-demanding tasks, particularly under stressful situations (Munoz *et al*., 2013; Robison *et al*., 2017; Tseng and Poppenk, 2020).

The clinical relevance of this personality trait (i.e. neuroticism) and the complexity of its multiple facets have generated great interest in the study of its neural correlates (Ormel *et al*., 2013a; Servaas *et al*., 2013). Research on the neurobiological basis of neuroticism has generally employed two main approaches. First, some studies investigated the correlation between neuroticism, brain morphology and brain connectivity at rest, based on the assumption that neuroticism is a semi-stable personality trait and should be reflected in semi-stable brain characteristics (Pang *et al*., 2016; Gentili *et al*., 2017; Schultz *et al*., 2017; Hsu *et al*., 2018; Ueda *et al*., 2018). Second, research has focused on the emotional distress component and considered the relationship between neuroticism and individual differences in neural responses to negative emotional stimuli (Canli *et al*., 2001; Cremers *et al*., 2010; Klamer *et al*., 2017). Altogether, these studies have found a consistent association of neuroticism with specific brain regions responsible for emotional and self-referential processing such as the amygdala and the ventromedial prefrontal cortex (Cremers *et al*., 2010; Schultz *et al*., 2017), but also with functional connectivity within large-scale brain networks (Gentili *et al*., 2017; Nostro *et al*., 2018). Yet, previous literature neglected how neuroticism affects the temporal dynamics of intrinsic brain networks and their reactivity to emotional events.

Here, we investigate the relationship between neuroticism and the temporal dynamics of resting-state networks associated with attention and self-referential thinking by employing microstates analysis of high-density EEG during recovery of negative emotional experiences. Microstates analysis is a spatio-temporal methodology for multichannel EEG that detects recurrent scalp potential topographies (i.e. microstates). Remarkably, the microstates topographies and temporal parameters that characterize spontaneous brain activity at rest are consistent across subjects and studies. Recent research suggests that four microstates classes (A, B, C and D) explain up to 80% of the variance in resting brain activity (for a review see Michel and Koenig, 2018), even though a few other, less prominent classes are also observed in some conditions (Custo *et al*., 2017). These EEG microstates reflect stable configurations of underlying neural networks and have been proposed as a promising electrophysiological signatures of fMRI-defined resting-state networks, offering a measure of their functional dynamics at the much higher time resolution provided by EEG (Britz *et al*., 2010; Custo *et al*., 2017). Specifically, class A has been associated with an auditory network, class B with a visual network, class C with both the salience network (SN) and default mode network (DMN), and class D with the attentional network (Britz *et al*., 2010; Custo *et al*., 2017). Furthermore, resting-state microstates have recently been employed to identify novel markers of specific psychopathologies, such as mood and anxiety disorders, depression, schizophrenia, or panic disorder (Kikuchi *et al*., 2011; Tomescu *et al*., 2015; Al Zoubi *et al*., 2019; Damborská *et al*., 2019). Among these psychopathologies, several are associated with high neuroticism, and microstate analysis might therefore also offer new insight on its neurobiological underpinnings.

In the present study, we instructed participants to rest after watching fearful or neutral video clips in order to investigate the relationship between neuroticism, spontaneous brain activity, and emotional regulation. Exposure to either negative or positive emotional events can cause long-lasting changes in spontaneous brain activity and connectivity during subsequent resting-state periods (Eryilmaz *et al*., 2011, 2014; Gaviria *et al*., 2021). This form of ‘affective inertia’ is sensitive to individual emotion regulation abilities and is abnormally prolonged in psychopathologies characterized by negative affect and ruminations (Kuppens *et al*., 2010, 2012; Baez-Lugo *et al*., 2023). We hypothesized that negative events should impact spontaneous EEG dynamics at rest during the post-emotion recovery periods, and that neuroticism would modify such effects. Specifically, given that a primary function of fear is to enable the organism to face external dangers and threats (Adolphs, 2013; Tracy, 2014), exposure to fearful events could induce a shift in the balance between internally- and externally-oriented processing modes at rest. Since microstate C has been associated with a portion of the default mode network responsible of self-referential thoughts (Panda *et al*., 2016; Michel and Koenig, 2018), we expected to observe a decrease of microstate C’s temporal parameters in favor of attention-related microstate D subsequent to fearful events.

Previous research has shown a great association between neuroticism and sustained self-generated thoughts that can perturb attentional processes (Robison *et al*., 2017). Thus, we hypothesized a positive correlation between neuroticism and temporal parameters of microstate C, but a negative correlation with microstates D. Finally, we predicted neuroticism to more globally affect the individual fear responses observed in the microstates’ temporal parameters.

## Materials and methods

### Subjects

A total of 36 individuals (11 male) participated in the experiment. Inclusion criteria were the absence of history of neurological or psychiatric disorders, age between 20 and 30 years (mean = 23.22, SD = 2.56), being right-handed and normal or corrected to normal vision. All participants signed a consent form approved by the local ethics committee and received compensation in the form of Swiss Francs (20.- CHF). Data were collected in accordance with the Declaration of Helsinki and the study protocol was approved by the local ethics committee.

### Experimental design

The experiment was implemented in Matlab using the Psychophysics Toolbox (Brainard, 1997) and consisted of 12 recording epochs. Each epoch started with a short video clip (mean duration = 51.6 s) followed by two beep sounds separated by an interval of 45 s of resting state. During this time frame, subjects were instructed to close their eyes and let their mind freely wonder.

The video clips used consisted of edited popular movies or TV pieces characterized by fearful or neutral content and were selected from the database previously employed by Eryilmaz and colleagues (Eryilmaz *et al*., 2011). Their emotionality ratings were validated in the same study, showing the absence of significant differences in emotional intensity ratings and low-level video features but reliable differences in their emotional valence. The neutral video clips were considered as an emotional ‘baseline’ condition in order to measure the impact of fearful clips on subsequent resting state and highlight the distinctive effect of emotional recovery during rest (Eryilmaz *et al*., 2011).

Resting-state epochs were assigned to two conditions according to the emotional content of the clip presented at their beginning (i.e. fearful and neutral). The number of epochs was balanced with respect to their emotional content and on each experimental session the video clips order was randomized.

After the experimental session participants were asked to fill the big five inventory (BFI), a 44-item inventory that measures an individual on the Big Five Factors of personality (extraversion, agreeableness, conscientiousness, neuroticism, and openness to experience) (John and Srivastava, 1999). Here, only the eight items regarding neuroticism were considered to compute each participant’s score (ranging from 8 to 40).

### EEG acquisition and pre-processing

The EEG was recorded using a high density (256 electrodes) HydroCel Geodesic Sensor Net (Electrical Geodesics Inc, Eugene, USA), sampled online at 1 kHz between DC and 100 Hz with a vertex reference. Electrodes on the cheeks and nape were excluded and the remaining 204 electrodes were kept for further analysis. The signal was pre-processed using a DC filter, a Kaiser band-pass filter (1–40 Hz) and a notch filter (50 Hz). Oculomotor and cardiac artifacts were removed using an InfoMax-based Independent Component Analysis (ICA) approach (Makeig *et al*., 1996). Bad electrodes were interpolated using a 3D spherical spline (Perrin *et al*., 1989) and noisy time segments were marked and excluded from successive analyses. On average 3.76% of data contained artifacts across participants (SD = 3.86, range = 0.26–19.03), rejected for analysis, but no participants were excluded. Finally, all channels were recomputed to the common average reference and EEG data were epoched in order to obtain a dataset for the resting-state epochs, starting at the offset of the initial beep sound of each trial and lasting 45 s.

All the pre-processing steps were computed using Brainstorm (Tadel *et al*., 2011), which is documented and freely available cfor download online under the GNU general public license.

### Microstates analysis

The free academic software Cartool (brainmapping.unige.ch/cartool) was used for the EEG microstate analysis (Brunet *et al*., 2011). In the following, we describe which processing steps were performed.

First, resting-state epochs were down sampled to 200 Hz and the Global Field Power (GFP) was computed. The GFP is a measure of the instantaneous potential variance across the electrodes, calculated as the standard deviation of the electrodes at a given time point and it is intended as a measure of topographical strength or signal-to-noise ratio (Brunet *et al*., 2011; Khanna *et al*., 2015).

Second, topographies located at the local maxima of the GFP were submitted to a modified version of the k-means cluster algorithm (Pascual-Marqui *et al*., 1995). The optimal number of clusters maps was determined by computing the best trade-off between the number of clusters used (ranging from 1 to 12) and the variance explained.

Importantly, the cluster analysis was performed first at the individual level in order to obtain two sets of representative maps for each subject (i.e. one per condition). This procedure was then repeated at the group level by employing the previously detected maps to identify the most representative microstates classes across subjects for each condition. The clustering was performed using the re-sampling methodology provided by Cartool to increase the reliability of the results. Previously detected short segments of noisy activity were ignored at this stage.

Third, the two sets of microstates classes were fitted to the original data using a ‘winner-takes-all’ criterion based on the spatial correlation between the identified maps and the time-series of each subject. Finally, the information provided by the microstates time-series was synthetized in two parameters:

• Mean Duration: the average length of a specific class of microstates, which measures the temporal stability of EEG-defined resting-state networks.

• Time Coverage: the percentage of the total recording time in which a microstate class dominates, which provides an index of the overall activation of the underlying network.

### Statistical analysis

To verify the presence of the same microstates classes in the fear and neutral conditions, therefore allowing for a direct comparison between their temporal parameters, we paired the classes between conditions according to their highest spatial correlation and employed a topographical ANOVA (TANOVA) (Habermann *et al*., 2018). This is a nonparametric method for the statistical comparison of EEG topographies, which we performed for each paired classes as implemented in the Cartool software.

Next, we performed a two-way repeated measures ANOVA (Microstate Classes × Conditions) for each temporal parameter. Given a significant interaction effect, we compared the temporal parameters (mean duration and time coverage) of each microstate class between the two conditions by means of multiple paired *t*-test (two-tailed). As a control analysis for a potential effect of gender on microstates temporal parameters, we recomputed the ANOVA and included gender as a nested factor. Results were Bonferroni corrected for multiple comparisons (α = 0.003).

Finally, we computed Pearson correlation coefficients between the neuroticism scores of the BFI and microstates parameters. First, we correlated the neuroticism scores and the parameters of each condition and second, we correlated the neuroticism scores with the significative microstates differences between conditions. Correlations results were corrected using False Discovery Rate.

## Results

**Fig. 1.**
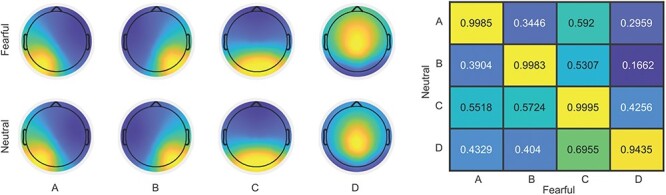
Results of the microstates analysis. (Left) Topographies of the four distinct microstates classes identified for the fearful and neutral conditions. (Right) Correlation matrix for topographies from the fearful and neutral conditions, entries indicate correlation coefficients.

The microstate analysis identified four representative microstate maps across subjects in the fear and the neutral conditions, which explained ∼68% of the total variance in the data (68.36% in the fear condition and 68.14% in the neutral condition). A visual inspection of these microstate topographies led to the recognition of the four canonical microstates classes (A, B, C and D) in both conditions, as previously reported (Michel and Koenig, 2018). This result was further confirmed by the highest spatial correlation between these topographies compared across the two rest conditions (see Figure 1). Moreover, the TANOVA failed to detect significant differences between the paired classes (A *P* = 0.26, B *P* = 0.14, C *P* = 0.28, D *P* = 0.70).

**Fig. 2.**
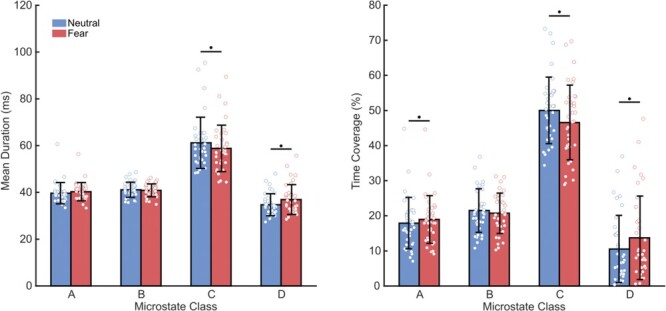
Group-level statistics for the microstates parameters. (Left) Mean duration of each microstates class × condition. (Right) Time coverage of each microstates class × condition. Error bars represent the standard deviation, asterisks indicate significant differences, Bonferroni corrected (eight comparisons).

The comparisons between microstate parameters of the fear and neutral conditions yielded highly significant results for classes C and D, and a marginal difference for class A. Specifically, the 4 (class) × 2 (emotion) ANOVA revealed a significant interaction effect for both the time coverage [*F*(3, 35) = 29.35, *P* < 10^−13^] and the mean duration parameters [*F*(3, 35) = 25.35, *P* < 10^−11^]. Subsequent *t*-tests showed that mean duration and time coverage of class C were significantly lower [*t*(35) = 4.35, *P* < 10^−3^, Cohen’s *d* = 0.22; *t*(35) = 5.72, *P* < 10^−4^, Cohen’s *d* = 0.34; two-tailed] in the fear condition with respect to the neutral condition; while for class D they were higher [*t*(35) = −6.13, *P* < 10^−5^, Cohen’s *d* = 0.29; *t*(35) = −6.77, *P* < 10^−6^, Cohen’s *d* = 0.39; two-tailed]. Finally, the time coverage of microstate A was also slightly greater after fearful movies [*t*(35) = −3.38, *P* = 0.014, Cohen’s *d* = 0.14; two-tailed] (see Figure 2). Notably, the microstates parameters across conditions were highly correlated for all the maps (all *P* < 0.001). Within-condition microstates reliability was also measured using the split-half method on the first and second half of each trial, separately for fearful and neutral trials. This demonstrated high internal consistency for all maps and temporal parameters in both conditions (all *r* > 0.70), confirming previous reports on the reliability of these measures (Khanna *et al*., 2014; Liu *et al*., 2020).

To control for a potential effect of gender on microstates temporal parameters, we included it as a nested factor in the ANOVA. These analyses showed significant (*P* < 0.05) interactions between microstate maps and gender (shorter microstates C and D parameters in male participants), corroborating the influence of gender differences on microstates (Tomescu *et al*., 2018; Zanesco *et al*., 2020). The interaction between maps and emotional condition still had a significant effect on all microstates’ parameters (all *P* < 0.05), indicating that post-emotional changes in resting EEG were globally similar in male and female participants.

**Fig. 3.**
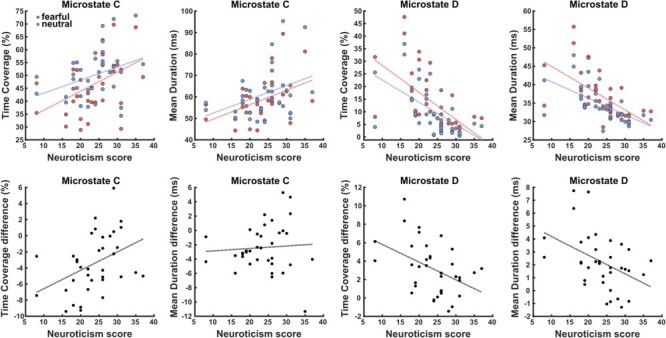
Relationship between neuroticism and microstates parameters across emotion conditions. (Top) Correlations between neuroticism scores and the parameters of classes C and D. Each scatter plot shows the parameters and regression lines for both the fearful and neutral conditions (red *vs* blue data points, respectively). (Bottom) Correlations between neuroticism scores and the emotion-related differences between conditions (fearful minus neutral).

Next, we examined the correlation between neuroticism and microstates parameters across classes and conditions. Neuroticism internal reliability was strong (*r* = 0.80, odd-even split-half method). Results showed significant effects for the temporal parameters of microstates classes C and D, but no correlation for other classes. In the fear condition, neuroticism scores exhibited a moderate positive relationship with the parameters of class C [mean duration: *r*(34) = 0.43, *P* = 0.013; time coverage: *r*(34) = 0.44, *P* = 0.015] and a strong negative relationship with the parameters of class D [mean duration: *r*(34) = −0.59, *P* < 10^−3^; time coverage: *r*(34) = −0.57, *P* < 10^−3^]. In the neutral condition, there was a similar correlation of neuroticism with the class C parameters [mean duration: *r*(34) = 0.37, *P* = 0.029; time coverage: *r*(34) = 0.34, *P* = 0.04], and again a negative correlation with class D [mean duration: *r*(34)= −0.60, *P* = 0.001; time coverage: *r*(34)= −0.59, *P* < 10^−3^]. In other words, participants with low neuroticism scores had lower class C and greater class D parameters at rest as compared with more neurotic participants, regardless of previous emotional stimulation (see Figure 3).

Finally, we investigated whether neuroticism scores were associated with a distinctive fear response in the post-emotional resting periods. We computed the difference between the fearful and neutral condition for each significant temporal parameter (decreases for class C, increases for class D and increases for class A), and then correlated these effects with neuroticism. This analysis further confirmed highly significant correlations positive for differences in time coverage of class C [*r*(34) = 0.40, *P* = 0.018] and negative for differences in both parameters of class D [mean duration: *r*(34) = −0.42, *P* = 0.013; time coverage: *r*(34) = −0.43, *P* = 0.015]. No significant correlation was found for class A (all *P* > 0.26). In other words, participants with lower neuroticism scores showed more pronounced fear-induced effects on resting-state microstates, while participants with higher neuroticism scores showed fear effects that were almost absent or even slightly in the opposite direction (see Figure 3B). As a further control analysis taking into account any effect of gender, for each correlation mentioned earlier, we computed a linear-mixed model including gender as a random intercept effect. Results were consistent with significant (*P* < 0.05) independent effects of gender and neuroticism on microstates parameters.

## Discussion

We investigated the neural underpinnings of neuroticism and its impact on emotional reactivity by means of topographic EEG analyses. Our goal was to shed light on the activity of large-scale brain networks at rest while harnessing the fine-grained temporal resolution provided by EEG to probe for their dynamic fluctuations after exposure to negative emotional events. Specifically, we built upon recent evidence that transient emotions can produce lasting changes in subsequent resting state, which reflect spontaneous recovery processes following stressful experiences (Eryilmaz *et al*., 2011; Gaviria *et al*., 2021). Our results uncovered a distinctive influence of neuroticism on the dynamic brain activity patterns associated with emotion regulation and stress resilience.

### Fear modulates microstates temporal dynamics

To the best of our knowledge, this is the first study to investigate affective responses at rest by means of EEG microstates analysis. We found that exposure to fearful video clips induces a decrease of the stability and presence of microstate C, accompanied by an increase in the presence of microstate D during subsequent resting state. There was a minor increase in microstate A and no effect on microstate B. A lasting influence of previous stimuli or tasks on resting-state brain activity was previously shown with fMRI. Feng and colleagues exploited this phenomenon to study the neural correlates of fear extinction after aversive conditioning (Feng *et al*., 2013, 2015, 2016). Eryilmaz and colleagues employed an experimental design similar to ours and found that previous fear-eliciting movies led to reduced fMRI activity in regions associated with the default mode network [i.e. anterior cingulate cortex (ACC), insula, precuneus and prefrontal cortex], together with lower frequency of self-referential thinking at rest (Eryilmaz *et al*., 2011). Likewise, Gaviria *et al*. (2021) found alterations in DMN dynamics at rest after sad movies (higher occurrence rate with no duration change), predicting their impact on subjective affect. Baez-Lugo *et al*. (2023) also reported a persistent emotion-induced increase in functional connectivity between posterior DMN (PCC) and amygdala, which correlated with negative thoughts and ruminations in a large cohort of healthy elderly individuals. In keeping with these observations, we found a consistent decrease of the temporal parameters of microstate C, a brain configuration pattern previously linked to introspective processes and to the activity of the default mode network (Britz *et al*., 2010; Custo *et al*., 2017; Bréchet *et al*., 2019). In contrast, we found an increased presence of the attention-related microstate D. This in turn fits well with the enhancement of attentional processes induced by fearful states (Phelps *et al*., 2006; Pourtois *et al*., 2013).

We propose that these changes may reflect a fear-induced shift toward an externally oriented processing mode at rest. Indeed, many studies reported enhanced attention and vigilance following fearful stimuli with more rapid and efficient processing of visual stimuli (Vuilleumier, 2005; Vuilleumier and Driver, 2007). Moreover, similar modulations of classes C and D have been reported when comparing the microstates dynamics of externally oriented brain modes (i.e. eyes-open resting state or attention-demanding tasks) with those of eyes-closed resting state (Seitzman *et al*., 2017; Bréchet *et al*., 2019; Zanesco *et al*., 2020).

Post-emotion increases were also observed in the time coverage of class A microstate, but not in its mean duration, and with much smaller effects than other changes. We had no prediction concerning this microstate A, previously associated with auditory networks (Michel and Koenig, 2018), and speculate that it might reflect increased auditory attention during closed-eyes resting due to heightened attention and vigilance after negative emotions. We also note that Kikuchi and colleagues found a similar increase in microstate A parameters in patients with panic disorder compared to controls during eye-closed resting state (Kikuchi *et al*., 2011). These results suggest that microstate class A might play a specific role in the regulation of negative emotions and associated arousal states (Kikuchi *et al*., 2011).

Taken together, our data provide novel evidence that emotional events can produce carry-over effects on brain state dynamics as measured in spontaneous EEG fluctuations, presumably reflecting an ‘affective inertia’ associated with the spontaneous emotion regulation and adaptive processes mediating the recovery from stressful events (Kuppens *et al*., 2010). In turn, these results further point to a key role in such processes for networks overlapping with DMN (and possibly SN), previously associated with class C microstates (Britz *et al*., 2010; Custo *et al*., 2017).

### Neuroticism’s influence over fear responses

Remarkably, we found that neuroticism scores correlated positively with the magnitude of fear-induced changes of class C and negatively with changes of class D. Thus, individuals with low neuroticism scores showed a stronger emotional carry-over effect on resting-state dynamics (i.e. heightened class D and reduced class C parameters, see Figure 3). While this result may appear at first sight to stand in contradiction to the notion that neuroticism magnifies negative emotional responses (Suls and Martin, 2005; Ormel *et al*., 2013a), it could actually fit well with a less effective and less flexible engagement of dynamic neural processes underlying emotion regulation and recovery from stressful events. Moreover, this interpretation would accord with the theory that a predominant and fixed focus on self-generated thoughts is the ‘engine’ of neuroticism (Perkins *et al*., 2015). Accordingly, Bréchet and colleagues found greater class C and lesser class D during both rest and autobiographical memory relative to externally driven cognitive (mental arithmetic) operations (Bréchet *et al*., 2019). In this light, the correlations observed here do not imply that neuroticism acts as an emotional flattener, but rather that it might alter the nature of emotional responses with a bias toward an introspective mode. This bias would attenuate the exteroceptive attentional benefits of fear and amplify the subjective negative feelings evoked by stressful situations, eventually contributing to the persistence of negative self-reflective thoughts and ruminations (Suls *et al*., 1998). This would also be consistent with previous evidence that for high neurotic individuals, stressful events might trigger a maladaptive coping style based on introspective processing which results in less attentional resources and prolonged negative feeling (Servaas *et al*., 2014). Hence, neuroticism might be associated with a greater imbalance of class C and D networks, regardless of previous stimulation (i.e. even in the baseline/ neutral condition) and thus less malleable during emotion recovery periods.

### Neuroticism and microstates classes

Neuroticism describes a stable cognitive and affective mode that favors the emergence of psychopathology. Accordingly, defining individual differences in post-stimulation resting microstates dynamics might provide a powerful and ‘controlled’ tool to better characterize the neural underpinnings of particular psychopathological traits. Our results support this idea, by showing that neuroticism scores correlate positively with the presence and stability of microstate C, and negatively with the parameters of microstate D. Remarkably, we found the same general correlations in the fear and neutral conditions, consistent with the presence of stable brain dynamic states at rest in neurotics, independent of transient emotional events, even though the magnitude of correlations was modulated by the negative movie condition. These results suggest that neuroticism may enhance the activity of neural networks involved in introspective thinking at the expenses of attentional networks.

Converging behavioral evidence corroborates this interpretation. Indeed, neurotic individuals are characterized by a tendency toward not only introspective processes such as rumination, mind-wandering and worry, but also poor performances in attention-demanding tasks (Munoz *et al*., 2013; Servaas *et al*., 2014; Robison *et al*., 2017). Accordingly, neuroimaging studies have found negative correlations between neuroticism and recruitment of attentional networks (Simon *et al*., 2020), as well as positive correlations with fronto-limbic regions involved in self-referential processing and appraisal of salient affective stimuli (Ueda *et al*., 2018). Higher rumination tendency (Baez-Lugo *et al*., 2023) and negative affect (Gaviria *et al*., 2021) in healthy participants were also found to correlate with more pronounced carry-over effects (inertia) in functional connectivity between DMN and limbic regions in fMRI resting state after exposure to negative videos.

Only a few recent studies investigated the relationship between neuroticism and EEG microstates dynamics. Zanesco and colleagues reported similar correlations between neuroticism and specific microstates (Zanesco *et al*., 2020). Notably, our findings align with existing research indicating that neuroticism and oxytocin have opposite effects on stress responses and, more generally, brain dynamics (Heinrichs *et al*., 2003). Schiller and colleagues found a positive effect of oxytocin administration on the temporal stability (i.e. mean duration) of all microstate classes (Schiller *et al*., 2019). This effect was more pronounced in individuals with high neuroticism scores, emphasizing the contrasting impact of oxytocin and neuroticism on stress responses (Heinrichs *et al*., 2003). Interestingly, in the same study, the administration of oxytocin produced a selective effect on microstates time coverage, namely a reduction of microstate C and an increase of microstate D (Schiller *et al*., 2019). This contrasts with our findings on neuroticism, where we observed an increase in class C and a decrease in D. In other words, these findings point to the divergent effects of oxytocin and neuroticism on stress responses, with oxytocin reducing and neuroticism enhancing these responses, as evidenced by changes in EEG microstates.

We believe our results have a more general relevance with respect to the association of neuroticism with schizophrenia (Ramanathan, 1984; Van Os and Jones, 2001; Ohi *et al*., 2016). Several studies examined microstates in schizophrenic patients and reported EEG changes similar to those we observed for high neurotic individuals: increased microstate C and reduced microstate D parameters (Tomescu *et al*., 2015; Rieger *et al*., 2016). This distinctive pattern has been associated with the severity of schizophrenic symptoms (i.e. hallucinations) (Kindler *et al*., 2011; Tomescu *et al*., 2014). This may point to a more fundamental implication of corresponding brain network dynamics in the development of psychopathology across particular diagnostic conditions. Moreover, it is likely that microstate patterns in EEG might emerge from the functional dynamics of widely distributed brain areas, involving more than a single network, and thus subsume different reciprocally interacting circuits depending on affective or cognitive conditions (Gaviria *et al*., 2021), with class C maps reflecting not only DMN activity but also contributions of other networks such as saliency or interoception-related networks (Custo *et al*., 2017).

Further, our data also corroborate the antagonistic functional nature of classes C and D and their involvement in a balance between attentional and introspective processes that could represent a crucial disturbance linked to higher risk of psychopathology (Rieger *et al*., 2016; Schiller *et al*., 2019).

On a final note, this study has some potential limitations. Although our experimental design was randomized, we cannot exclude that our finding may be influenced by cumulating carry-over effects of successive emotional clips, or conversely modified by progressive habituation to negative stimuli. Additionally, our sample consisted mostly of young students, had a relatively limited size, and was not balanced with respect to gender (see ‘Materials and methods’ section). Our auxiliary analysis controlling for gender and split-half comparisons support the reliability of the relation observed with neuroticism and its generalization to both males and females, but some correlations among personality scores may be volatile and require large samples or replication to be definitely established (Pace and Brannick, 2010). Future research should investigate in more detail not only the effects of gender but also ageing and other demographic features on the relationship between microstates and neuroticism. In any case, our data provide novel evidence for emotion-induced effects on resting state EEG and interindividual differences therein.

## Conclusions

Our findings are based on a novel application of the microstate analysis in the domain of affective neurosciences and further support the potential clinical value of this method. The analysis of resting-state activity and its modulation by emotional events shed new light on the neural underpinnings of fear responses and an important facet of neuroticism, that is the tendency to engage in self-generated thoughts (Perkins *et al*., 2015) and its detrimental impact on attentional control (Robison *et al*., 2017). Highly neurotic individuals showed greater class C parameters and lower class D parameters compared to individuals with lower neuroticism scores, but smaller changes with respect to the aforementioned pattern in the aftermath of fearful stimulation. These effects were independent, but modulated by emotional context. We interpret our results as useful markers of emotion regulation abilities and difficulties of neurotic individuals to disengage from introspective processes in order to enable more efficient attention and vigilance. These abnormal dynamics may represent a common neural mechanism responsible for psychopathology in different disease conditions.

## Data Availability

Data and code are available on github (https://github.com/DeoMiche/NeuroStates).
